# Arsenic exposure associated T cell proliferation, smoking, and vitamin D in Bangladeshi men and women

**DOI:** 10.1371/journal.pone.0234965

**Published:** 2020-06-23

**Authors:** Scott W. Burchiel, Fredine T. Lauer, Pam Factor-Litvak, Xinhua Liu, Tariqul Islam, Mahbubul Eunus, M. Abu Horayara, Md. Tariqul Islam, Mizanour Rahman, Alauddin Ahmed, Serge Cremers, Renu Nandakumar, Habibul Ahsan, Christopher Olopade, Joseph Graziano, Faruque Parvez

**Affiliations:** 1 Department of Pharmaceutical Sciences, College of Pharmacy, University of New Mexico, Albuquerque, NM, United States of America; 2 Department of Epidemiology, Mailman School of Public Health, Columbia University, New York, NY, United States of America; 3 Department of Biostatistics, Mailman School of Public Health, Columbia University, New York, NY, United States of America; 4 University of Chicago and Columbia University Field Research Office, Dhaka, Bangladesh; 5 Department of Pathology and Cell Biology, Columbia University Medical Center, New York, NY, United States of America; 6 Irving Institute for Clinical and Translational Research, Columbia University Medical Center, New York, NY, United States of America; 7 Department of Health Studies, University of Chicago, Chicago, IL, United States of America; 8 University of Chicago Medical Center, University of Chicago, Chicago, IL, United States of America; 9 Department of Environmental Health Sciences, Mailman School of Public Health, Columbia University, New York, NY, United States of America; University of Texas Medical Branch at Galveston, UNITED STATES

## Abstract

There are limited data examining the consequences of environmental exposure to arsenic on the immune system in adults, particularly among smokers. Smoking has been shown to exacerbate or contribute to impaired immune function in men chronically exposed to arsenic. In contrast, vitamin D (VitD) is known to have a positive influence on innate and adaptive immune responses. The effect of circulating VitD on arsenic-associated immune dysfunction is not known. Here we examine the relationship of arsenic exposure and T cell proliferation (TCP), a measure of immune responsiveness, and circulating VitD among adult men and women in Bangladesh. Arsenic exposure was assessed using total urinary arsenic as well as urinary arsenic metabolites all adjusted for urinary creatinine. TCP was measured *ex vivo* in cryopreserved peripheral blood mononuclear cells from 614 adult participants enrolled in the Bangladesh Health Effects of Arsenic Longitudinal Study; serum VitD was also evaluated. The influence of cigarette smoking on arsenic-induced TCP modulation was assessed only in males as there was an inadequate number of female smokers. These studies show that arsenic suppressed TCP in males. The association was significantly strong in male smokers and to a lesser extent in male non-smokers. Interestingly, we found a strong protective effect of high/sufficient serum VitD levels on TCP among non-smoking males. Furthermore, among male smokers with low serum VitD (⊔20 ng/ml), we found a strong suppression of TCP by arsenic. On the other hand, high VitD (>20 ng/ml) was found to attenuate effects of arsenic on TCP among male-smokers. Overall, we found a strong protective effect of VitD, when serum levels were >20 ng/ml, on arsenic-induced inhibition of TCP in men, irrespective of smoking status. To our knowledge this is the first large study of immune function in healthy adult males and females with a history of chronic arsenic exposure.

## Introduction

Environmental exposure to arsenic has many health effects in chronically exposed populations [1, 2]. Surprisingly, there have been only a few population-based studies that have examined the effects of arsenic on the immune system of humans. These studies, all with relatively small sample sizes, have focused on PBMC obtained from adults and children [3, 4], or on cord blood leukocytes [5, 6]. The sole study on adults was conducted among individuals with arsenic induced skin lesions, an indirect measure of arsenic exposure [3].

Previous work from our labs has shown that arsenic modulates various immune functions measured in cryopreserved peripheral blood mononuclear cells (PBMC) obtained from men chronically exposed to arsenic in Bangladesh [7, 8]. Those studies were limited to 181 males equally divided between smokers and never smokers as well as low and high arsenic exposure. We measured TCP in stimulated PBMC, but we did not find any significant associations with arsenic.

In the present study, we recruited 614 smoking and non-smoking adults from the Health Effects of Arsenic Longitudinal Study (HEALS) cohort in Bangladesh. The current study has a cross-sectional design. Historically, the HEALS participants were chronically exposed to arsenic, though mitigation strategies have reduced exposure during the past decade [9]. PBMC were isolated from the blood samples and assayed for TCP by using two mitogens: anti-CD3/CD28 and phytohemagglutinin (PHA) as well as no mitogen (to measure unstimulated proliferation). Previous *ex vivo* studies have revealed differences between the effects of *in vitro* exposures to arsenic for these two mitogens [10]. In the present study we also investigated the role of serum VitD on TCP, as VitD has been associated with immune status and modulation in past population studies [11, 12]. To our knowledge, there has not been any study that examined TCP in a large healthy population with individual assessment of multiple measures of arsenic exposure.

## Methods

### Study population

Prior to the start of this study, ethical clearance was obtained for the study protocol from the Bangladesh Medical Research Council and was approved by Columbia University’s Institutional Review Board. To ensure that the translation of the consent forms and recruitment materials were accurate they were translated into Bengali and back translated into English. A village health worker from the area was made available for any person unable to read the informed consent or requiring explanation of procedures. Each participant provided either verbal or written consent in the presence of a witness. The protocol for analyses of the biological samples was approved by the Health Science Center’s Human Rights Protection Office of the University of New Mexico.

Healthy men and women between the ages of 35 and 65, regardless of smoking status, and living in the study area were eligible for this study. Using eligibility criteria, a list of 2,197 potential participants was generated from the HEALS central database. Initial steps in the recruitment process included a home visit by a field team. Upon the visit to the home potential participants were deemed ineligible due to the following: death (3), migration out of the study area (21), suffering from a serious or multiple chronic illnesses (52), illness or symptoms related to immune function disruption (48), taking medication(s) that might have an impact on immune function (27), and 341 were not at home. Our field team also found that 803 individuals were using a different source of drinking water (tube well) from what they had reported at the time of initial recruitment to the HEALS. Of the 902 eligible participants, 791 agreed and visited the study clinic whereas some eligible participants (19) missed their appointment due to conflict with their work schedule, or because their work location was out of the study area and they could visit the study clinic only over the weekends. There were no significant differences in age and sex between the individuals who agreed and refused to participate in the study. Blood and urine samples were collected from each participant. Hematology tests, such as full blood count and blood glucose levels were conducted. Twenty-five individuals were excluded due to: abnormal blood sugar levels (8), suffering from urinary tract infection (9) or lymphocytosis (2). Furthermore, three samples were excluded at time of PBMC isolation due to hemolysis and another three for low cell count and low viability. A total of 766 PBMC samples were shipped to University of New Mexico using dry nitrogen shippers. Upon thawing of PBMC, 147 samples were found to have viabilities less than 80% and four samples had low cell numbers and were not assayed for TCP. At the time of analysis, arsenic exposure data was missing for one sample. Thus, 614 samples were analyzed for TCP.

### Measurement of arsenic exposure

Urine samples (15 ml) were collected at the study clinic at Araihazar, Bangladesh. Total arsenic in urine was accessed by graphite furnace atomic-absorption spectrophotometry (GFAAS) as previously described [7, 8]. Urinary creatinine, quantified by a colorimetric method based on the Jaffe reaction, was used to correct urinary arsenic (UAs) and metabolites. All the exposure measures, including total urinary arsenic and metabolites were expressed as μg/g of creatinine.

### Collection and cryopreservation of peripheral blood

Approximately 10 ml of blood was collected at the field clinic by technicians proficient in blood collection. Detailed procedures [13] were followed for PBMC isolation, freezing, and storage. Dry shippers (Cryoport, Irvine, CA) that maintain the temperature at or below -150°C were used to ship samples from Bangladesh to the United States. Upon arrival samples were stored in liquid nitrogen until thawed. Samples were thawed quickly in a 37°C water bath. Cell counts and viabilities were acquired with a Nexcelom Cellometer Auto 2000 Cell Viability Counter using acridine orange and propidium iodide (AO/PI; Nexcelom Bioscience, CS2-0106) according to manufacturer’s directions. Cells with viabilities exceeding 80% were used for immune function testing.

### T cell proliferation assay

A standard mitogenesis assay using tritiated (3H) thymidine previously described [7] was used to access T lymphocyte proliferation. Briefly, cells were plated into six replicate wells at 1x10^5^ cells/wells (for each mitogen) in a 96 well, flat bottom tissue culture plates. PBMC were then stimulated with each mitogen; anti-CD3 antibody (0.5 μg/ml (in DPBS) [clone OKT3 functional grade eBiosciences, 16-0037-85]) and anti-CD28 antibody (2 μg/ml per well [clone CD28.2 functional grade eBiosciences, 16-0289-85], PHA (5μg/ml per well [phytohemagglutinin-M from *phaseolus vulgaris*] Sigma Millipore 11082132001) or media as a “no stimulation” control (to evaluate background stimulation). Plates containing cells and mitogen were incubated for 72 hr in a humidified incubator with 5% CO_2_ at 37˚C. Following incubation, cultures were pulsed with 1 μCi/well ^3^H thymidine and returned to the incubator for overnight incubation (16–18 hr). Individual wells were harvested onto angel hair filters using a Brandel 96 well harvester (Gaithersburg, MD). Filters were air-dried for at least 1.5 hr at RT, then placed into scintillation vials containing scintillation fluid. A Beckman Coulter LS6500 Multipurpose Scintillation Counter was used to count each sample for 1.5 min. The data is reported here as counts per minute (CPM).

### Measurement of serum 25-hydroxyvitamin D concentrations

Sample serum 25-hydroxyvitamin D (the sum of D_2_ and D_3_) levels were assayed in the Department of Medicine at the Columbia University Medical Center. 25-hydroxyvitamin D_2_ (ergocalciferol; 25(OH)D_2_) and 25-hydroxyvitamin D_3_ (cholecalciferol; 25(OH)D_3_) were measured using Ultra-Performance Liquid Chromatography- mass spectrometry (LCMS/MS) as described previously [14, 15]. Briefly, 25(OH)D_2_ and D_3_ were extracted from human serum samples using liquid–liquid extraction. LCMS analysis was done using a triple quadrupole Agilent 6410 (Agilent, Santa Clara, CA) mass spectrometer. Chromatographic separation was performed on a Poroshell 120 EC-C18 column (3.0 x 50mm, 2.7 μm) using a gradient of 70%-90% methanol containing 0.1% formic acid. The mass spectrometer was operated under multiple reaction monitoring (MRM) mode with positive electrospray ionization with the following MRM transitions: 413->395 for 25(OH)D_2_, 401->383 for 25(OH)D_3_ and 407->389 for d6-25(OH)D_3_. Lower limit of quantitation for the assay for both 25(OH)D_2_ and 25(OH)D_3_ was 1.0 ng/ml. Intra-day precision was 2.4% for 25(OH)D_2_ and 3.5% for 25(OH)D_3_. Inter-day precision was 8.1% for 25(OH)D_2_ and 5.5% for 25(OH)D_3_. Calibrators were standardized against the NIST standards and the assay passed the proficiency testing of international DEQAS. Calibrators are standardized against the NIST standards and the laboratory participated in the international DEQAS proficiency scheme. In the U.S. the normal reference range for total 25-hydroxyvitamin D (25(OH)D) is 30–100 ng/mL [16].

### Statistical analysis

We excluded 7 smoking women from analyses. The final dataset included a total of 607 samples. To describe the sample characteristics, we calculated percent for categorical variables and means for quantitative variables. ANOVA test was used to detect group differences, especially for the variables included in TCP, serum VitD, and total UAs and UAs metabolites (Inorganic arsenic (InAs; arsenite and arsenate), monomethylarsonic acid (MMA) and dimethylarsinic acid (DMA)) adjusted for urinary creatinine. Spearman correlation coefficients were used to describe bivariate associations among these quantitative variables. Scatterplots were used to describe the preliminary relationships between the exposures of interest (e.g. UAs and UAs metabolites) with T-cell function stimulated by anti CD3/CD28, and thus inform the specification of statistical model.

We used generalized additive models (GAM) to evaluate possible non-monotonic relationships between T-cell proliferation, as the outcome, and UAs as well as the metabolites, as the exposures. GAM allowed for both a parametric component of exposure (which assesses a linear relationship) and non-parametric components, which assesses non-monotonicity.

We fit linear regression models for all T-cell function outcomes using each an exposure variable as the primary predictor; all models were adjusted for age and body mass index (BMI). We stratified regression models by smoking status and sex (not for women, there were only 7 smokers). To reduce the impact of extreme variables and improve model fitting, we transformed the exposure variables with right skewed distributions with the logarithmic function. We extended our analyses by adding serum VitD concentrations into the linear regression models. To test whether VitD status modified the associations between arsenic and T-cell function, we stratified VitD into two groups with low/deficient serum levels (⊔20 ng/ml) and high/sufficient levels (>20 ng/ml) and tested the interaction between vitD status and arsenic.

SAS version 9.4 was used for statistical analysis. R version 3.5.1 was used to create figures for this manuscript.

## Results

### Characteristics of the study population

A total of 614 individuals were enrolled in this study, of which 392 were non-smokers and 222 were smokers; 48.2% were men and 51.8% women (Table 1). The average age of study population was 50 years; male smokers were slightly older (53 years) than non-smoking males (52 years) and females (47 years). There were only 7 female smokers out of a total of 222 smokers (3.15%); therefore, it was not possible to compare the effects of smoking on TCP in men and women and we excluded smoking women from all analyses. The average total urinary arsenic concentrations across samples was 135 μg/g urinary creatinine and did not differ between smokers, non-smoking males, or non-smoking females. However, the levels of MMA, were significantly higher among male smokers compared to non-smoker males and females. Similarly, the serum level of VitD was also higher among male smokers than non-smoker males and females. In these studies, we found that some men and women were VitD deficient, but they were otherwise generally healthy. This provided us with an opportunity to assess immune function (TCP) in Vit D sufficient and deficient men and women. BMI levels in male smokers were lower than those of non-smoking men and women.

**Table 1:** Sample characteristics.

	All samples (n = 614)	Non-smoking Women (n = 311)	Non-smoking Men (n = 81)	Smoking Men (n = 215)	p-value
**Demographic**					
Age (years)	50	47	52	53	p<0.0001
Women (%)	51.80				
BMI	23.02	24.02	22.50	21.81	p<0.0001
Smoking (%)		50.65	13.19	35.01	
**Average Arsenic Exposure Conc. (μg/g)**					
Urinary arsenic	134. 78	140.75	105.08	137.26	
Inorganic arsenic	13.83	13.23	10.67	15.84	
MMA	16.46	15.12	12.09	19.94	p = 0.001
DMA	86.79	91.69	68.26	86.68	
**Proliferation (counts per minute)**					
CD3-CD28	105,763.5	109,147.5	100,742.7	102,543	p<0.0001
PHA	66,407.1	67,831.5	63,114.3	65,130.5	
No-stimulation	996.80	985.5	834.7	1073.3	
**Vitamin D (ng/ml)**	22.65	19.28	25.64	26.43	p<0.0001

p-values were from ANOVA test for differences in quantitative variables among three groups (smoking men and non-smoking men and women)

### Effect of arsenic on TCP

Among all the study participants (n = 607), we observed negative associations between all measures of arsenic exposure and TCP activated with anti-CD3/CD28 in models adjusting for age and BMI (Table 2). We did not observe an effect of arsenic on TCP among non-smoking women. We observed similar point estimates of the associations in the male smoking and non-smoking groups but results were only statistically significant in the male smokers. In the male smokers (n = 215), we observed a strong negative effect on TCP for all arsenic exposure measures (p<0.05) in adjusted models. We conducted analysis to check for an interaction between arsenic and smoking, no interaction was detected.

**Table 2:** Estimated coefficient of arsenic exposure in linear models for CD3-CD28 stimulated T cell proliferation.

	All participants (n = 607)	Non-smoking Women (n = 311)	Non-smoking Men (n = 81)	Smoking Men (n = 215)
**Exposure**^a^				
Urinary arsenic	-1495.4 (-3312.3, 321.3)	243.4(-2422.1, 2909.0)	-3645.4 (-008.0,1717.1)	-2972.0 (-5786.3, -157.7)*
Inorganic arsenic	-948.8 (-2506.7,609.0)	861.5 (-1391.5,3114.7)	-2172.6 (-6707.9,-362.7)	-3048.6 (-5506.7,-590.5)**
MMA	-764.6 (-2322.1,792.8)	594.2(-1693.1, 2881.6)	-1183.8 (-946.1,3578.4)	-2559.5 (-4960.3,-158.6)*
DMA	-1597.7 (-3411.5, 215.9)	246.4 (-2399.0, 2891.9)	-3476.2 (-684.3,1731.8)	-3234.9 (-6076.8,-393.0)*

^*a*^ Linear regression models were run separately for different arsenic exposure measures (log transformed) and adjusted for age and BMI; Values are B (95% confidence Intervals) p-values:

*p < .05,

**p<0.01

### Protective effect of vitamin D on arsenic induced TCP

**Fig 1:**
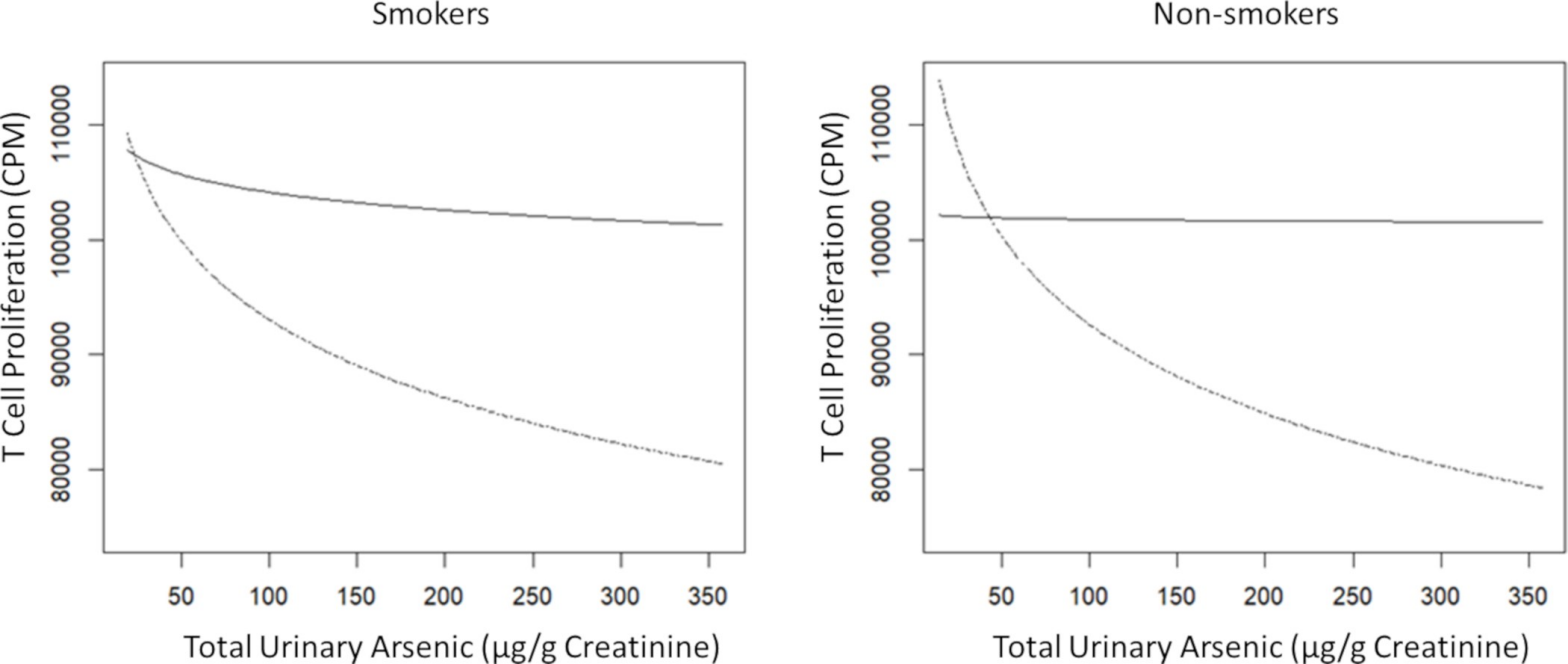
Age and BMI adjusted association of urinary arsenic with T cell proliferation (TCP) in male smokers and non-smokers with sufficient or deficient serum VitD levels. Male Smoker (n = 173) and Male Non-smoker (n = 61) by High/sufficient VitD serum concentration (>20 ng/ml), indicated by black solid line, and low/deficient serum VitD (<20 ng/ml) by broken line. Approximately 20% of male smokers and non-smokers in both groups were found to be VitD–deficient.

In models with the additional predictor of VitD, we did not observe any significant changes in the arsenic and TCP associations. However, VitD was positively associated with TCP for all groups and strongly associated in males, both smoking and non-smoking (Table 3). Among non-smoking males compared to smoking males, the positive associations between vitD and TCP was stronger, regardless of the As exposure variable used in the models (p<0.05). When stratifying VitD into low/deficient and high/sufficient levels (Table 4) we did not see significant changes in the associations of arsenic with TCP in non-smoking women. However, among non-smoking males with low/deficient VitD, arsenic was found to significantly suppress TCP (p<0.05) (Table 4). In smoking males with low/deficient VitD level the inhibition of TCP by arsenic exposure remained significant (p<0.05). In contrast, high/sufficient VitD found to significantly attenuate effect of arsenic on TCP in smoking men such that As exposure becomes a non-statistically significant predictor. A binary depiction of the relationship between total urinary arsenic and TCP by VitD strata in male smokers and non-smokers is shown in Fig 1.

**Table 3:** Estimated coefficients of arsenic exposure and Vitamin D (VitD) in linear models for CD3-CD28 stimulated T-cell proliferation.

	All participants (n = 607)	Non-smoking Women (n = 311)	Non-smoking Men (n = 81)	Smoking Men (n = 215)
**Exposure**^a^				
Urinary arsenic^b^	-1760.2 (-3581.6, 61.2)*	121.5 (-2596.8, 2839.8)	-3775.5 (-7747.9, 196.8)	-2999.6 (-5896.1,-103.0)*
VitD	341.2 (117.5, 564.4)**	248.6 (-97.1, 594.4)	689.71 (127.2, 1252.1)**	310.99 (-41.0, 663.0)
InAs^b^	-1088.5(-2646.2,469.2)	884.8 (-1418.8, 3188.5)	-1659.0(-6082.8,764.8)	-3036.7(-5564.0, -509.5)**
VitD	326.3 (102.9, 549.7)**	229.1 (-116.3, 574.6)	651.1 (86.2, 1216.1)*	308.2 (-41.1, 657.6)
MMA^b^	-1043.2(-2610.2, 523.7)	460.6 (-1891.3, 2812.6)	-1044.5(-5691.8, 3602.7)	-2683.4(-5180.0, -186.8)*
VitD	337.7 (113.1, 562.2)**	239.9 (-107.6, 587.4)	664.5 (97.4, 1231.6)*	325.5 (-27.1, 678.2)
DMA^b^	-1950.5(-3774.3, 26.7)*	100.4 (-2465.1, 2666.0)	-3441.4(-8601.9, 1719.0)	-3462.5(-6394.6, -530.3)*
VitD	350.2 (126.1, 574.2)**	248.7 (-97.9, 595.3)	692.8 (129.9, 1255.7)**	334.97 (-17.4, 687.3)

^**a**^ Linear regression models were run separately for different arsenic exposure measures (log transformed) and are adjusted for age, BMI and VitD; Values are B (95% confidence Intervals); p-values:

*<0.05,

**<0.01

^b^ Arsenic exposure measures were log-transformed

**Table 4:** Estimated coefficient of arsenic exposure in linear models for CD3-CD28 stimulated T-cell proliferation by low and high vitamin D (VitD) levels.

	All participants (n = 607)	Non-smoking Women (n = 311)	Non-smoking Men (n = 81)	Smoking Men (n = 215)
**Arsenic Exposure**^a^ **and Vitamin D**^b^				
**Urinary arsenic**				
Low VitD	-778.9 (-4014.2, 2456.3)	2115.5 (-1674.5, 5905.6)	-11196.0 (-18193.1, -4199.0)**	-9871.9 (-17703.8, -2039.9)*
High VitD	-1197.9 (-3410.4, 1014.5)	-1859.7 (-5610.4, 1890.8)	-184.7(-7205.3,6835.8)	-2244.0 (-5108.7,620.6)
**InAs**				
Low VitD	-1206.3 (-3868.6, 1455.9)	1153.6 (-1851.8, 4159.0)	-8451.4 (-14429.4, -2473.4)*	-8005.6 (-14820.5, -1190.7)*
High VitD	-722.5 (-2689.1, 1244.1)	508.4 (-2919.4, 3936.2)	1448.4 (-4483.1, 7380.0)	-2583.7(-5088.1,-79.3)*
**MMA**				
Low VitD	-746.9 (-3488.5, 1994.7)	1558.2 (-1592.8, 4709.3)	-8339.4 (-15114.1, -1564.6)*	-6803.0 (-13480.7,-125.3)*
High VitD	-915.7 (-2855.5, 1024.0)	-771.3 (-4146.7, 2603.9)	2211.3 (-3857.9, 8280.6)	-2304.0 (-4749.6, 141.5)
**DMA**				
Low VitD	-1042.1 (-4295.5, 2211.1)	1502.5 (-2226.9, 5232.0)	-11199.1 (-18051.9, -4346.4)**	-11147.4 (-19554.6,-2740.1)**
High VitD	-1210.6 (-3412.7, 991.3)	-1325.3 (-5101.0, 2450.3)	-351.7 (-7194.5, 6491.0)	-2632.7 (-5471.5, 205.9)

^a^ Linear regression models were run separately for different arsenic exposure measures (log transformed) adjusted for age and BMI; Values are B (95% confidence interval); p-values: p < .05*, p < .01**

^*b*^ VitD level [low/deficient: ⊔20 ng/ml; high/sufficient: >20 ng/ml]

### Phytohemagglutinin (PHA) induced TCP was not inhibited by arsenic

We previously found that activation of TCP by PHA was more sensitive than anti-CD3/CD28 activated TCP during *in vitro* exposure of PBMC to MMA^+3^ [10]. Therefore, we compared PBMC stimulated with anti-CD3/CD28 with those stimulated with PHA. We found that PHA-induced TCP was not associated with total UAs, InAs, MMA, or DMA in male or female regardless of smoking status (S1 Table). Additionally, we found that baseline proliferation of PBMC in the absence of mitogen activation was not sensitive to arsenic or metabolite exposures (S2 Table). Therefore, anti-CD3/CD28 appears to be an appropriate T cell mitogen to assess the effects of arsenic on TCP from donors exposed *in vivo*.

## Discussion

Arsenic has complex effects on immune responses measured in animal models and human lymphoid cells exposed *in vitro* [17]. One of the difficulties in assessing immune effects of arsenic in human populations is that multiple functional assays must be performed to measure effects on the myriad of immune mechanisms associated with innate and adaptive immunity. Various measures of adaptive immunity [7, 10, 18–21] and innate immunity [22–24] are suppressed by arsenic exposure.

TCP is critical for cell activation, effector, and helper cell functions [25]. TCP is a simple measure of immune function that has previously been used in in a few population-based studies to study arsenic related immunomodulation [3, 4]. In the present study, we found that total UAs, as well as InAs, MMA, and DMA were associated with a decrease in anti-CD3/CD28 stimulated TCP in smoking males, and to a lesser extent in male non-smokers but not females. We did not observe any effects of arsenic on non-smoking women, although they were exposed to similar level. We are unaware of any study that examined effects of arsenic on TCP by smoking status or sex. An earlier study of 38 adults did not examine effects of arsenic on TCP by gender or smoking status (Biswas 2008).

In this study, we did not find an effect of arsenic on PHA- stimulated TCP. The result differs from our previous work, where we described PHA-stimulated TCP as being more sensitive to MMA^+3^; however, the past study was performed *in vitro* among 30 donors with much lower arsenic concentrations [10].

The mechanism(s) associated with arsenic immunosuppression are likely due to a combination of genotoxic and non-genotoxic actions on lymphoid cells. The consequences of immunosuppression produced by arsenic include an increased susceptibility to infections [3, 24, 26, 27]. The genotoxic actions of arsenic are likely due to increased DNA damage and oxidative stress [21, 28–32]. The non-genotoxic actions of arsenic are associated with altered signaling pathways [33, 34].

In non-smoking males with low serum VitD, arsenic exposure was associated with a suppression of TCP (p<0.02). On the other hand, smoking men with high serum VitD were found to have a noticeably reduced association between arsenic and TCP. The findings clearly demonstrate a harmful effect of low serum VitD and a beneficial effect of high serum VitD on TCP in arsenic exposed males. However, there was no association seen in females. Overall levels of VitD were higher in males than females, presumably due to their outside work and sun exposure [35]. Males also had slightly higher levels of urinary arsenic, perhaps due to increased consumption of drinking water or arsenic exposure in cigarettes. Because we observed important differences between arsenic-induced immunosuppression in males and females, it is important to examine potential mechanisms responsible for these observations. 1,25-dihyroxyvitamin D_3_, the biologically active form, is a well-known modulator of T cell function [11, 12, 36, 37] and T cell development [38, 39]. VitD receptors (VDR) are known to be expressed on human T cells, and they play a role in T cell activation [40]. Heterogeneity in VitD responses may be due to VDR polymorphisms [41–43]. VitD therapy has been attempted for certain autoimmune diseases [44–46], based on results in animal models showing that VitD can increase the ratio of Treg to Th17 cells [47, 48]. During the past decade there have been numerous clinical trials to restore immune health in people exposed to HIV [49, 50] and other infectious diseases [51] using VitD supplementation.

A limitation of the study is that effects of smoking on arsenic induced TCP were assessed among males only because only a few number of females were smokers. Other studies will need to be conducted to determine whether there are sex differences in smoking and non-smoking populations.

T cells are one of the most important components of the adaptive immune system. They are essential for an adaptive immune system and immune response. T cells play a major role in protecting against many adverse health outcomes. Low T-cell counts or inhibition of functional activity may increase the risk of intracellular pathogens such as viruses, protozoa and intracellular bacteria, and in immunity to extracellular pathogens by providing help for the antibody response. It is unclear why the TCP responses to arsenic differed between men and women in the current study. It is well known that arsenic metabolism involves methylation, which is associated with homocysteine and folate pathways that significantly differ in men and women [52, 53]. Therefore, the sex-related differences in immunosuppression produced in males and females may in part be due to altered metabolism of arsenic.

The public health significance of this work relate to the following observations. Our findings demonstrate that T cell responses to arsenic differ by sex, men being more susceptible, particularly smokers. We show that VitD may be an important modulator of immune responses. VitD levels significantly modified effects of arsenic on TCP; high VitD was protective and low VitD was harmful, which were only apparent in men. Our results suggest in arsenic exposed populations, smoking cessation and VitD supplementation might be beneficial for T cell function and subsequent health effects.

## Supporting information

S1 Table Estimated coefficient of arsenic exposure in linear models for PHA stimulated T cell proliferation. (PDF)

S2 Table Estimated coefficient of arsenic exposure in linear models for non-stimulated T cell proliferation. (PDF)
