# Supplementary material for: Arsenic exposure associated T cell proliferation, smoking, and vitamin D in Bangladeshi men and women
Source: PLoS One. 2020 Jun 23;15(6):e0234965. doi: 10.1371/journal.pone.0234965 (PMC7310686; doi:10.1371/journal.pone.0234965)
Supplement: S1 Table — (PDF) [file pone.0234965.s001.pdf]

**Supplemental Table 1: Estimated coefficient of arsenic exposure in linear models for PHA stimulated T cell proliferation**

| | **All samples** | **Non-smoking women** | **Non-smoking men** | **Smoking men** |
|---|---|---|---|---|
| **Exposure**[a] | B | B | B | B |
| Urinary As | 0.004 | 0.063 | -0.131 | -0.033 |
| Inorganic As | -0.006 | -0.096 | 0.021 | -0.029 |
| MMA | 0.021 | -0.087 | 0.075 | -0.024 |
| DMA | 0.0004 | -0.165 | 0.083 | -0.051 |

[a] Linear regression models were run separately for different arsenic exposure measures (log transformed) adjusted for age and BMI.
