# Supplementary material for: Arsenic exposure associated T cell proliferation, smoking, and vitamin D in Bangladeshi men and women
Source: PLoS One. 2020 Jun 23;15(6):e0234965. doi: 10.1371/journal.pone.0234965 (PMC7310686; doi:10.1371/journal.pone.0234965)
Supplement: S2 Table — (PDF) [file pone.0234965.s002.pdf]

S2 Table: Estimated coefficient of arsenic exposure in linear models for non-stimulated T cell proliferation

	**All samples**	**Non-smoking women**	**Non-smoking Men**	**Smoking Men**
**Exposure**[a]	B	B	B	B
Urinary As	0.014	0.038	-0.012	-0.018
Inorganic As	-0.001	0.018	-0.002	-0.048
MMA	0.018	0.036	0.002	-0.016
DMA	-0.001	0.025	-0.015	-0.041

[a] Linear regression models were run separately for different arsenic exposure measures (log transformed) adjusted for age and BMI.
